# An evaluation of school-based e-cigarette control policies’ impact on the use of vaping products

**DOI:** 10.18332/tid/93594

**Published:** 2018-08-22

**Authors:** Sandra Milicic, Philip DeCicca, Emmanuelle Pierard, Scott T. Leatherdale

**Affiliations:** 1 University of Waterloo, Waterloo, Canada; 2 McMaster University, Hamilton, Canada

**Keywords:** youth, electronic cigarettes, school-based policies and programs, interventional evaluation, natural experiment

## Abstract

**INTRODUCTION:**

Electronic cigarette (e-cigarette) use among youth is common, and so efforts to regulate its use and availability are continually being made. The school environment represents an important domain for advancing health policy among youth populations. This study examines the impact of school-based e-cigarette control policies on student e-cigarette use in the context of a natural experiment.

**METHODS:**

Using three years of longitudinal student and school level data (2013/2014 to 2015/2016), from a sample of 69 secondary schools in Ontario, Canada, a generalized estimating equation approach examined the impact of school-based e-cigarette control policy changes on the prevalence of youth e-cigarette use. The main outcome of interest was current e-cigarette use, while covariates included age, gender, ethnicity, and amount of spending money in dollars per week the student has. Tests of proportion (t-tests) were used to examine whether there were any significant differences in the changes for each intervention school relative to the sample of schools that report no changes in school-level e-cigarette control policies.

**RESULTS:**

Estimates from the generalized estimating equation approach suggest that students had lower odds of using e-cigarettes in schools where an e-cigarette control policy was implemented. That is, the e-cigarette control policy decreased the adjusted odds of being an e-cigarette user (OR=0.68; 95% CI: 0.48–0.97). Examining school-specific impact, at four of six schools that had an e-cigarette control policy, the ban on the use of e-cigarettes may have lowered the prevalence of e-cigarette use.

**CONCLUSIONS:**

This is the first study to use longitudinal data to study school-level e-cigarette use and the impact of e-cigarette control policy. These results provide new evidence that school-level policies banning the use of e-cigarettes on school property may be effective in reducing e-cigarette use (or preventing it) in their current form, as seen in this natural experiment.

## INTRODUCTION

Electronic cigarettes (e-cigarettes) were introduced to markets in China in 2004, and have rapidly expanded globally^1^ and increased in popularity. In Canada, about 18% of grade 6–12 students reported having ever tried an e-cigarette and 6% reported use in the past 30-days in 2014/2015^2^. In Ontario and Alberta, there was a 35% relative increase in current use of e-cigarettes by grade 9–12 students from 7.2% in 2013/2014^3^ to 9.7% in 2014/2015^4^. In the US, past month use of e-cigarettes by 10th and 12th graders was about 14% in 2015 and 11% in 2016^5^, and exceeded past 30-day cigarette use in those years^5^. E-cigarette use has been linked with combustible cigarette use: youth cigarette smokers have a higher prevalence of e-cigarette use relative to non-smokers^3,6,7^ and youth e-cigarette users are more susceptible to start smoking^8,9^. E-cigarette use has the potential to renormalize smoking^10,11^ and the prevalence of past 30-day use of e-cigarettes among non-smokers is increasing^12^.

E-cigarettes are marketed as a healthier alternative to smoking traditional cigarettes^13-15^, and that they can potentially act as a smoking cessation aid^16-18^. Evidence suggests that while e-cigarettes are not without health risks, they are likely to be far less harmful than conventional cigarettes since they contain fewer and lower levels of toxicants than conventional cigarettes^19^. However, while e-cigarette aerosol may contain fewer toxicants than cigarette smoke, studies evaluating the health consequences of long-term use of e-cigarettes are inconclusive and incomplete^19-22^. The lack of clear evidence on the health effects of e-cigarettes^23^ coupled with the upward trend in e-cigarette use is particularly worrisome for the health of youth, since brain development continues throughout adolescence and into young adulthood^24,25^. While e-cigarettes may be a less harmful alternative to cigarette smoking, they are not harmless^23^. Given the increased uptake and growing markets of e-cigarettes, regulatory approaches and public policy interventions are warranted, as the literature on the effects of e-cigarettes is in its infancy.

Recent Federal leadership in both the US and Canada have complemented State and Province efforts to regulate e-cigarette use and availability. In 2018, the Government of Canada introduced new legislation, the Tobacco and Vaping Products Act^26^, which amends the Tobacco Act to regulate vaping products (i.e. e-cigarettes) as a separate class of products. One of the aims of this amendment is regulation aimed to make e-cigarettes less accessible to youth, thereby protecting Canadians from nicotine addiction and tobacco use^26^. Similarly, in 2016 the US Food and Drug Administration finalized a rule extending their regulatory authority to cover all tobacco products including e-cigarettes. It regulates the manufacture, import, packaging, labeling, advertising, promotion, sale and distribution of all electronic nicotine delivery systems^27^. The school environment represents an important domain for advancing health policy among youth populations. Not only do most youth (regardless of socioeconomic status) spend approximately 25 hours each week in school throughout the school year but also there is substantial evidence in the tobacco control literature demonstrating that the school policy environment can represent an important context for shaping youth tobacco use behavior^28-30^. While legislation on tobacco product use on school grounds, and elsewhere, has existed for a long time in Ontario, legislation on the use of e-cigarettes in general is more recent. Youth have been favourably affected by legislation related to tobacco, and school bans on e-cigarette could have a similar impact on the use of vaping products in youth.

To date, there is a gap in the evidence of the impact of school-based e-cigarette control policies on use of e-cigarettes by youth. Within the context of a natural experiment, using a generalized estimating equation (GEE) approach, the current study evaluates the impact of school-based policies banning the use of e-cigarettes at school on youth e-cigarette use. The data provide robust and easily interpretable results of the impact that e-cigarette controls policies in secondary schools have on subsequent youth e-cigarette use. The results of this natural experiment give this study the unique opportunity to inform the literature and policy related to youth e-cigarette use and prevention.

## METHODS

### Data

The COMPASS Study is an established school-based system designed to effectively guide and improve youth prevention research and practice^31^. It was designed to collect hierarchical longitudinal data from a cohort of secondary school students in grades 9 to 12 and the schools they attend in Ontario and Alberta, Canada. The current study utilized linked longitudinal student-level data from a sample of 69 Ontario secondary schools that participated in three waves of the COMPASS host study when e-cigarette data were collected for three ‘school years’ [Y2 (2013/2014), Y3 (2014/2015) and Y4 (2015/2016)]. Alberta secondary schools in the COMPASS host study were excluded since municipal by-laws govern sale and use of e-cigarettes in Alberta. Y1 (2012/2013) data were excluded from the sample since questions related to youth e-cigarette use were introduced in Y2. A full description of the COMPASS host study and its methods are available in print^31^ or online (www.compass.uwaterloo.ca). All procedures were approved by the University of Waterloo Office of Research Ethics and appropriate school board committees.

### Participants

School boards and schools were purposefully selected based on whether they permitted active-information passive-consent parental permission protocols^31^, crucial for collecting robust data among youth pertaining to substance use^32^. Eligible schools were approached after board granted approval. Students could decline to participate at any time. Missing respondents resulted primarily from scheduled spares (mostly an issue for grade 12 students) or absenteeism during data collection.

To explore longitudinal changes among respondents, Y2, Y3 and Y4 student-level data were linked within the 69 schools. The process of linking the student data across waves is described in more detail elsewhere^33^. Due to the rolling sample design^31^, it was not possible to link the grade 12 students in Y2 (or Y3) who graduated and thus would not have attended the school in Y3 (or Y4). Similarly, it was not possible to link the grade 9 students that were newly admitted to participating schools in Y3 (or Y4) and did not attend the school in Y2 (or Y3). The other main reasons for non-linkage included students transferring schools or dropping out, students not providing data for grade or gender in either year, students on spare or absent during data collection, or inaccurate data provided in the linkage measures. A total of 8363 Ontario secondary school students were successfully linked between Y2, Y3 and Y4, by matching students with their unique identification numbers_34_.

### Data collection tools

The student-level questionnaire for COMPASS (Cq) collects individual student data pertaining to multiple behavioral domains (e.g. marijuana use, eating behavior, tobacco use, physical activity, etc.), correlates of the behaviors, and demographic characteristics. In each school, the Cq was used to collect within-school samples during class time. The Cq items were based on national standards or current national public health guidelines, as described elsewhere^31^.

Changes to the provision of school-based e-cigarette policies within the schools between Y2 and Y3, and Y3 and Y4 were measured using the COMPASS School Programs and Policies Questionnaire (SPP). The SPP is a paper-based survey completed annually by the school administrator(s) most knowledgeable about the school program and policy environment within a school. The SPP measures the presence or absence of relevant programs and/or policies, changes to school policies, practices, or resources that relate to student health. The completed SPP from each school was collected by COMPASS staff at the time of their school’s student-level data collection. COMPASS staff also follow up with each school to verify the information provided.

### Measures

#### Student-level measures

Current use of e-cigarettes, is modeled using the following question: ‘In the last 30 days, did you use any of the following? (Mark all that apply). Options are: pipe tobacco, cigarillos or little cigars, cigars, roll-your-own cigarettes, loose tobacco mixed with marijuana, e-cigarettes, smokeless tobacco, nicotine patches, nicotine gum, nicotine lozenges, or nicotine inhalers, hookah to smoke tobacco, hookah to smoke herbal sheesha/shisha, blunt wraps, haven’t used any of these things in the last 30 days’. Consistent with previous research, respondents who reported using e-cigarettes in the past 30 days were considered current e-cigarette users^3^.

Controls for age (≤14, 15, 16, 17, ≥18 years), gender (female, male), ethnicity (‘White’, ‘Black’, ‘Asian’, ‘off-reserve Indigenous’, ‘Latin American/Hispanic’, ‘Other/Mixed’) and amount of spending money in dollars per week (zero, 1–20, 21–100, >100) were included as correlates in the model to account for omitted variable bias.

#### School-level measures

By law^1^, individuals cannot use lighted tobacco products inside a school or on school property in Ontario. Changes to school e-cigarette control programs and policies were identified using the SPP administered in Y3 and in Y4. The SPP asked administrators to report if there have been any changes to their school tobacco control practices and policies since the last school year. Between Y2 and Y3, three Ontario secondary schools reported implementing a control policy related to e-cigarettes and maintained their e-cigarette control policies into Y4. Between Y3 and Y4 three additional schools implemented e-cigarette control policies. The type of e-cigarette control policy implemented at all treatment schools was a ban on the use of e-cigarettes on school property/premises. These interventions were based on amendments made to current school-based tobacco control programs and policies at those secondary schools. The policies prohibit e-cigarette use on school property, in private vehicles parked at school, within a specific distance of the school and at sponsored events off school grounds.

### Sample construction and analyses

A total of 222 observations were removed for the following reasons. One secondary school that already had an e-cigarette control policy in Y1 was excluded (n=136 removed). Those for whom there was inconsistent response with the past 30 days of use of alternate tobacco products and cigarettes (n=86) were removed. To preserve sample size, the missing covariates were taken into account by creating binary variables [removed age (n=46), removed ethnicity (n=105) and removed spending money (n=293) including those who did not know how much spending money they had]. Note that excluding data with missing values (i.e. incomplete case analysis) did not change the resulting coefficient estimates or the interpretation of effects but the current design contributed to a more precise estimation. The final sample size was 8141 grade 9 to 12 students in 69 Ontario secondary schools that were followed over the three ‘school years’.

The generalized estimating equation (GEE) method was used to examine the marginal (population-averaged) effects of e-cigarette control policies on e-cigarette prevalence of youth use. The GEE accounts for correlated responses in panel data — that is, it accounts for the correlation induced by repeated collection of observations over time and/ or space^35^. Since e-cigarette use is a binary outcome, the GEE was modeled with a binomial distribution family, a logit link function, and an exchangeable (equal correlation among repeated measures) within-panel correlation structure to account for any correlation of students within school clusters. The resulting estimates were converted to odds ratios for ease of interpretation.

The treatment group comprised all schools that implemented an e-cigarette control policy between Y2 and Y3 and maintained the policy in place through to Y4, and schools that implemented an e-cigarette control policy between Y3 and Y4. The control group comprised schools that did not report implementing an e-cigarette control policy in Y3 or Y4.

Current smoking status was included in some estimations as a covariate while all other correlates were included in both models^2^. Some policy changes related to e-cigarette control may impact on smoking indirectly. Thus, estimating the models by including and excluding smoking status was a sensitivity check for the resulting estimates. Finally, tests of proportion (t-tests) were used to examine whether there were any significant differences in the changes for each intervention school relative to the sample of schools that reported no changes in school-level e-cigarette control policies. Stata version 14.2 was used in the analysis^36^.

## RESULTS

As shown in Table 1, in Y2 approximately 3.7% of the sample were considered current e-cigarette users and that increased to 7.6% in Y3 and to 8.8% in Y4. The ratio of males and females accounted for approximately the same proportion. Consistent with the underlying (Ontarian) population, the sample was predominantly ‘White’, followed by ‘mixed’ ethnicity, ‘Asian’, ‘Black’, ‘off-reserve Indigenous’, and ‘Latin America/Hispanic’. Most students in the sample had $1–$20 to spend per week or $20-$99 depending on the year. The vast majority of students in all years were non-smokers. Significant differences by gender for e-cigarette use were tested and documented by the significant chi-squared statistic.

**Table 1:** Descriptive statistics of the longitudinal sample of students from participating secondary schools in the COMPASS study Ontario, Canada for Year 2 (2013/2014), Year 3 (2014/2015), and Year 4 (2015/2016)

	*Year 2 (N=8141 )*		*Year 3 (N=8141 )*		*Year 4 (N=8141 )*		*p*
**E-cigarette use**	%	n	%	n	%	n	
Uses e-cigarettes	3.7	304	7.6	615	8.8	720	0.000
**Age, years**							
≤14	47.2	3846	2.3	191	0.0	1	0.021
15	42.0	3421	45.0	3666	2.5	204	0.000
16	10.0	814	42.1	3427	43.7	3561	0.120
17	0.5	42	9.8	801	42.4	3451	0.439
18	0.0	2	0.6	45	11.1	905	0.036
19	0.2	16	0.1	11	0.2	19	0.000
**Gender**							
Male	52.1	4245	52.1	4245	52.0	4231	
Female	47.5	3864	47.5	3864	47.3	3850	
Not reported	0.4	32	0.4	32	0.7	60	
**Ethnicity**							
White	76.9	6264	76.9	6263	76.0	6187	0.000
Black	3.4	277	3.5	281	3.7	302	0.000
Asian	5.7	462	5.6	457	5.9	484	0.576
Off-reserve Indigenous	1.5	121	1.6	133	1.6	131	0.681
Latin American/Hispanic	1.6	127	1.8	150	2.0	159	0.000
Other/Mixed	10.3	838	10.2	828	10.5	854	0.125
Missing	0.6	52	0.4	29	0.3	24	0.000
**Spending money ($/week)**							
zero	21.4	1745	17.5	1422	13.1	1067	0.000
1–20	39.5	3212	31.1	2534	19.9	1621	0.027
21–100	20.6	1678	26.1	2122	29.0	2361	0.000
>100	5.1	412	14.0	1143	27.6	2244	0.000
don't know, not stated	13.4	1094	11.3	920	10.4	848	0.000
**Smoking status**							
Current smoker	1.0	83	2.5	206	4.8	384	0.000
Former smoker	0.2	15	0.5	42	0.7	60	0.001
Non-smoker	98.8	8043	97.0	7893	94.5	7622	0.000

The p-value denotes where there are significant differences by gender for each covariate, as tested by the chi-squared statistic. There are N=8141 observations in each year as students that could be followed over the entire time of the study (3 school years) were only selected.

In Table 2, results from the GEE model show that the e-cigarette control policy had a statistically significant effect on e-cigarette prevalence. The e-cigarette control policy decreased the odds of being an e-cigarette user (OR=0.68; 95% CI: 0.48–0.97). Again, estimating models including and excluding smoking status as a control, represented a sensitivity check for the resulting estimates that suggests that estimates are marginally larger when smoking status is included in the model. Note that the magnitude of the relationship remains unchanged.

**Table 2:** Regression results from the Generalized Estimating Equation (GEE) of the effect of school bans on e-cigarette use prevalence

	*OR (Includes smoking as a covariate)*	*95% CI*	*p*	*OR (Excludes smoking as a covariate)*	*95% CI*	*p*
**E-cigarette use**						
Does not use (Ref)						
**Uses**	0.68	0.48–0.97	0.032	0.64	0.45–0.90	0.012

OR: odds ratio, CI: confidence interval. GEE analysis uses three years of the longitudinal sample of students from participating secondary schools in the COMPASS study in Ontario, Canada for Year 2 (2013/2014), Year 3 (2014/2015), and Year 4 (2015/2016).

**Figure 1:**
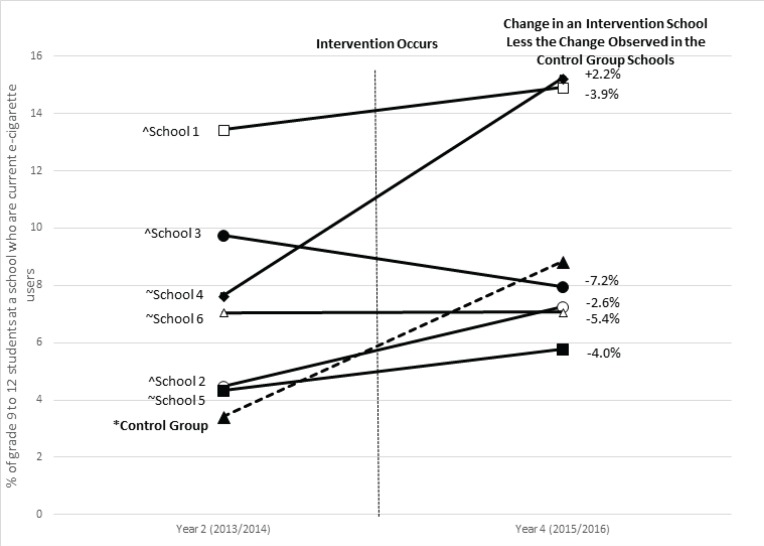
Results of the school-level e-cigarette control policies with pre-post differences in the school-level prevalence of current e-cigarette users. Data are from the longitudinal sample of students from participating secondary schools in the COMPASS study in Ontario, Canada for Year 2 ( 2013/2014 ), Year 3 ( 2014/2015 ), and Year 4 ( 2015/2016 ). ^ Indicates that a program or policy was implemented between Year 2 and Year 3; ~ Indicates that a program or policy was implemented between Year 3 and Year 4; *Denotes a statistically significant pre-post difference at p<0.05

The impact of e-cigarette control policies on individual schools is summarized in Figure 1. The results show that there was a statistically significant increase in e-cigarette use in control schools from 3.7% in Y2 to 8.8% in Y4. There were no robust mean difference changes in each of the treated schools. However, by visual inspection of the data — 4 of the 6 treatment schools had prevalence rates of e-cigarette use higher than control schools in Y2 and showed lower prevalence of e-cigarette use than control schools after the implementation of the policy. Schools 1 and 4 had higher prevalence of use than control schools before and after the ban. Therefore, for individual secondary schools, e-cigarette control policies may in fact have an impact on the prevalence of e-cigarette use. This confirms the results of the GEE.

## DISCUSSION

This study examined how e-cigarette control policies impact on youth use of e-cigarettes. Using a longitudinal student-level dataset over a three-year period with data from 69 secondary schools in Ontario, Canada, it appears that a ban on the use of e-cigarettes on school property may be effective in decreasing youth use of e-cigarettes or preventing youth use of e-cigarettes compared to schools that do not implement a school program or policy. The odds of reporting e-cigarette use at schools with a policy is significantly lower compared to schools without a policy. These results provide new evidence that school-level policies banning the use of e-cigarettes on school property may be effective in their current form, as seen in this natural experiment. Some research has found that the enforcement of policies has a positive impact on students adhering to policies^28^. COMPASS did not collect information on this. Based on the results from this study, future studies should explore the impact of enforcement of such policies.

The popularity of e-cigarettes^3,10,37–40^ coupled with the unknown long-term health effects^19–23^ and fear of renormalization of cigarette smoking^10,11,17,41^ is leading to stronger regulation of the product(s) and policies aimed at attenuating youth use of e-cigarettes. Both the United States and the Canadian Federal Governments have or are in the process of regulating the production and sale of e-cigarettes and implementing policies aimed at attenuating use^26,27^, while at the local level, States and Provinces are amending tobacco control policies to include e-cigarettes in the same class of products and thus follow the same legislation.

Many of the school-based interventions targeted to youth are not amenable to randomization at the school-level (i.e. policies or community-built environments) given that they are dictated at a regional, provincial (State) or national level. In such instances, researchers could take advantage of quasi-experimental designs for evaluating the natural experiments that occur as school stakeholders or policymakers implement different policies within school environments, or change community environments surrounding schools on an ongoing basis^42^. Given that e-cigarettes continue to gain popularity and use among youth, despite their uncertain long-term health effects, developing a better understanding of how school level policies may impact on e-cigarette use is an important consideration for school planners and policymakers. As such, this study is the first to use a natural experiment approach to examine how the ban on e-cigarette use on school property impacts on youth use of e-cigarettes.

Moreover, school policy and program behavior can act as a signal of concern if policies and programs are implemented or changed in order to address youth health-compromising behaviors. Over the time frame of analysis, 2013/2014 to 2015/2016, six Ontario schools enacted an e-cigarette control policy that banned the use of e-cigarettes on school property/premises. The statistical results suggest that these policies are promising and may have been effective in influencing youth e-cigarette use. In a study of the impact that tobacco control policies in COMPASS secondary schools have on tobacco use, the type of tobacco control policies implemented was important in considering its impact on youth use of cigarettes^28^. As such, it may serve school planners and policy makers well to consider the kind of e-cigarette control policy aimed at attenuating youth e-cigarette use to effectively address the concern. While most of the schools that implemented a ban had higher e-cigarette use rates than the control schools prior to the ban, it is likely that if the control schools were to implement such bans, a reduced use prevalence would likely be observed. It is however difficult to predict whether the magnitude of these effects would be similar to what we have measured here.

This is the first study to use longitudinal data to study school-level e-cigarette use and e-cigarette control policy. The research design thus has a strong internal validity because COMPASS data collected longitudinally at the student and school level minimizes systematic error while maintaining a strong external validity due to the quasi-experimental design. What is clear from this study is that e-cigarette control policies may in fact have an impact on youth use.

### Limitations

The current study is not without its limitations. Data collected on the use of e-cigarettes is self-reported, which may lead to an underreporting bias related to the use of e-cigarettes. Similarly, future studies would benefit from more detailed information related to the type, quantity and reason of use of e-cigarettes, or school-level information on the enforcement of school e-cigarette policies. There may be other relevant confounding factors that this study was not able to control for due to data limitations. For example, there is no information about the type of e-cigarette used by youth. Future studies using expanded COMPASS student data can examine the impact that this intervention has had on youth use of e-cigarettes. Lastly, new legislation banning sales of e-cigarettes to individuals under the age of 19 years came into law on 1 January 2016. The effects of this policy change will only become apparent once newer data from COMPASS become available.

## CONCLUSIONS

E-cigarette use is common among youth. There is substantial evidence in the tobacco control literature that demonstrates that the school policy environment can represent an important context for shaping youth tobacco use behavior. Using longitudinal school- and student-based data, this study evaluates the impact of e-cigarette control policies on subsequent e-cigarette use by youth. The results of this study provide new evidence that school-level policies banning the use of e-cigarettes on school property may be effective in reducing e-cigarette use (or preventing e-cigarette use) in their current form.

## CONFLICTS OF INTEREST

Authors have completed and submitted the ICMJE Form for Disclosure of Potential Conflicts of Interest and none was reported.
